# Sequential delay and probability discounting tasks in mice reveal anchoring effects partially attributable to decision noise

**DOI:** 10.1016/j.bbr.2022.113951

**Published:** 2022-06-02

**Authors:** Gerardo R. Rojas, Lisa S. Curry-Pochy, Cathy S. Chen, Abigail T. Heller, Nicola M. Grissom

**Affiliations:** Department of Psychology, University of Minnesota, 75 East River Rd, Minneapolis, MN 55455, USA

**Keywords:** Delay discounting, Probability discounting, Modeling, Touchscreen, Mice

## Abstract

Delay discounting and probability discounting decision making tasks in rodent models have high translational potential. However, it is unclear whether the discounted value of the large reward option is the main contributor to variability in animals’ choices in either task, which may limit translation to humans. Male and female mice underwent sessions of delay and probability discounting in sequence to assess how choice behavior adapts over experience with each task. To control for “anchoring” (persistent choices based on the initial delay or probability), mice experienced “Worsening” schedules where the large reward was offered under initially favorable conditions that became less favorable during testing, followed by “Improving” schedules where the large reward was offered under initially unfavorable conditions that improved over a session. During delay discounting, both male and female mice showed elimination of anchoring effects over training. In probability discounting, both sexes of mice continued to show some anchoring even after months of training. One possibility is that “noisy”, exploratory choices could contribute to these persistent anchoring effects, rather than constant fluctuations in value discounting. We fit choice behavior in individual animals using models that included both a value-based discounting parameter and a decision noise parameter that captured variability in choices deviating from value maximization. Changes in anchoring behavior over time were tracked by changes in both the value and decision noise parameters in delay discounting, but by the decision noise parameter in probability discounting. Exploratory decision making was also reflected in choice response times that tracked the degree of conflict caused by both uncertainty and temporal cost, but was not linked with differences in locomotor activity reflecting chamber exploration. Thus, variable discounting behavior in mice can result from changes in exploration of the decision options rather than changes in reward valuation.

## 1. Introduction

Delay discounting tasks measure value assessments against a temporal cost, while probability discounting tasks measure value assessments against risky reward [21,40]. These tasks have been important tools in assessing dysregulated reward processing in neuropsychiatric disorders such as addiction or neurodevelopmental disorders [12,2,44, 47]. Because of this, translational animal versions of these tasks are of high interest [35,54]. However, it is unclear if animals use similar discounting strategies to those used by humans [60], for two reasons.

One issue arises from the fact that behavior in each of these tasks alone are thought to reflect choice impulsivity in animals [1], even though these tasks contribute in different ways to assessing an impulsive profile [56]. It has recently been shown in humans that multiple distinct discounting tasks are needed to better capture common traits [5]; methods testing both delay and probability in the same animals are therefore of strong interest, but not widely available or used, especially in mice.

A second issue is that discounting tasks are typically modeled in both humans and rodents using economic value functions (e.g., *k*-values and *h*-values) which assume the main relevant factor in choices is the current discounted value of the reward [40]. However, recent evidence from the literature on reinforcement learning and decision making strongly implicates choice history and exploration as important variables in how both humans and animals perform value-based decision tasks [10,13,51, 8]. Importantly, animals often engage in non-reward seeking behaviors that are typically described as exploration [16,20]. This decision “noise” is rarely considered as a contributor to choices in discounting tasks despite exploratory events being necessary for animals to learn new task rules [14,15]. In humans, exploration in other decision making tasks correlates with the degree of delay discounting shown [48]. We have recently identified exploration as a key driver of sex differences in other decision making tasks [8,9]. Because discounting tasks are often used to compare groups of animals modeling neuropsychiatric risk factors or other individual differences such as sex differences [23,41,42,59,61], it is imperative to identify methods that allow us to distinguish whether differences in behavior are due to value judgments putatively reflecting impulsivity, or if exploration is a latent contributor to behavior.

One way to address these issues is to develop a method allowing within-subjects comparison of delay and probability discounting functions. This approach would allow for comparing overall performance within and between groups and permit computational modeling across tasks incorporating a decision noise parameter in addition to a value parameter. In the present study, we describe a sequence of delay and probability discounting tasks in mice achieving these goals. Mice are increasingly used for cognitive task batteries because of their high genetic tractability. Recent advancements in technology for mouse operant testing available through touchscreens have substantially improved the ease of training mice [27], enabling us to develop matched versions of probability and delay discounting for mice.

Here, we describe a novel battery of sequential delay and probability discounting schedules in touchscreens tested in male and female wildtype mice. One key factor previously shown to affect choices in these tasks is the order of presentation of delays or probabilities on the large reward (e.g. “Improving” or “Worsening” [30,32,53]). We alternated mice between Worsening and Improving schedules within each discounting task, and demonstrated that these order effects are substantial in both male and female mice. These order effects are fully eliminated in both sexes with extensive training in delay discounting. However, order effects remain in probability discounting, especially in female mice, consistent with sex differences in risk processing but not “impulsivity” per se [23]. We analyzed reward strategy via both win-stay/lose-shift analyses in probability discounting, and for both tasks with computational models incorporating both value and decision noise parameters in order to better understand how mice adapt choice strategies between delay and probability discounting. Win-stay/lose-shift analyses suggest schedule differences in probability discounting emerged because females learned to adjust win-stay behavior consistently over experience with probability discounting. Delay discounting is captured by the combination of a value and decision noise parameter, but probability discounting is better explained by the choice parameter. Analysis of choice response times and locomotor behavior suggest that reductions in exploratory choices with increased experience are linked with increased deliberation between choices, and are likely not due to simple changes in task engagement. These results demonstrate that value functions may capture one aspect of impulsivity (i.e. overall reward preference), but that exploratory or “noisy” decisions are significantly contributors to mouse behavior in these tasks, especially as choice behavior changes between tasks or across multiple experiences of the same task.

## 2. Materials and methods

### 2.1. Subjects

8 male and 7 female BL6129SF1/J mice (from Jackson Laboratories) took part in both delay discounting and probability discounting. Mice were housed in groups of 3–4 (2 groups of males, 2 groups of females). Mice began experiments at approximately 70 days of age. Mice had free access to water and were food restricted with their home chow at 85–90% of their baseline weight. Mice were pre-exposed to the operant reinforcer, vanilla flavored Ensure, in their home cage for one day prior to training. Ensure was freely available to be licked from a bottle. Each group of mice were verified to have consumed a full bottle of Ensure (148 ml). Behavioral testing took place Monday to Friday, and on Fridays, mice had free access to home chow. Animals were housed on a reverse light dark cycle (9 am-11 pm) and were tested during the dark period. Animals were cared for in accordance with National Institute of Health guidelines and were approved by the University of Minnesota Institutional Animal Care and Use Committee.

### 2.2. Apparatus

16 identical triangular touchscreen operant chambers (Lafayette Instrument Co., Lafayette, IN) were used for training and testing. The touchscreen was housed in the front while the food delivery magazine in the back. Information on individual touches on touchscreens throughout sessions were recorded via ABET-II software. Touchscreens were limited by masks with holes which allowed responding in 5 square holes. Liquid reinforcer (50% diluted Ensure) was pumped via a peristaltic pump (1000 ms or 250 ms duration, corresponding to volumes of approximately 25 μl and 6.25 μl). ABET-II software (Lafayette Instrument Co., Lafayette, IN) was used to program operant schedules and analyze all data from training and testing.

### 2.3. Behavioral procedures

#### Magazine training.

Mice received free 7 μl of Ensure every 30 s for 30 min in operant chambers for 5 days. Mice learned to approach the magazine to obtain Ensure.

#### Center Hole Fixed-Ratio 1.

Mice were initially trained to nosepoke the center hole of a 5-hole mask on the touchscreen chamber on a Fixed-Ratio 1 schedule for 10 days, 30 min each day. 7 μl of Ensure was delivered immediately following a nosepoke. Only the center hole was illuminated during these sessions. The magazine holding the Ensure was illuminated until mice interrupted an infrared beam when their head entered the reward port, and this allowed them to move to the next trial. All mice were moved on to the next phase once they could reach the max number of trials needed to complete delay discounting schedules (60 trials).

#### Chaining Center to Left and Right.

For this phase of training, hole 3 (center) illuminated and a nosepoke resulted in 7 μl of Ensure. After a center nosepoke and its reward, on the next trial, holes 2 (left) and 4 (right) illuminated, and mice learned that nosepoking either side resulted in a large amount (28 μl) of Ensure. Training lasted 29 days with 30 min sessions. Responses on holes 1 and 5 were counted as non-reinforced touches. Mice were moved on to the next schedule when they could consistently complete over 60 trials.

#### Responding on Sides Only.

Mice learned to nosepoke only the left and right holes for Ensure at the same volumes as above for 13 days. The left and right holes were the only holes to light up during these sessions. Center hole nosepokes no longer delivered the reinforcer. Sessions ended after 30 min had elapsed. Mice were moved on to the next schedule when they could complete 60 trials.

#### Responding on Chained Sides Followed by ITI.

Mice then learned to chain a center hole nosepoke to a left or right hole nosepoke for 13 days. The action sequence of center-to-left or center-to-right led to a large reward (25 μl) or small reward (6.25 μl). This phase of the testing was counterbalanced; half of the mice experienced the large reward on the left and the other half on the right. An inter-trial interval (ITI) of 30 s followed in order to suppress the reward rate. Animals were limited to no more than two trials in a row selecting one side before being forced to try the other side, to ensure they experienced the small reward side as well as the large reward. Sessions ended after 30 min had passed. Mice were moved on to the next schedule when they could complete 60 trials.

#### Improving and Worsening Delay Discounting.

To test the influence of anchoring effects, we tested mice on a Worsening schedule that was at the start of the session was initially favorable and became unfavorable as the session proceeded, then reversed the order of delays for the Improving schedule, then reversed again 4 additional times. We used the criteria set by Mar & Robbins [33] to judge if mice were ready to move on from the initial discounting round. We ran mice for extended periods of time (≥ 9 sessions) in order to ensure behavior had stabilized between rounds. Mice underwent 10 days of Worsening Delay Discounting I followed by 9 days of Improving Delay Discounting I, 9 days of Worsening Delay Discounting II and 11 days of Improving Delay Discounting II, and 18 days of Worsening Delay Discounting III and 18 days of Improving Delay Discounting III. One side delivered a large but delayed reward (25 μl) or a small and immediate reward (6.25 μl). Each delay block of trials began with 2 forced trials where mice had to choose the large delayed side in order to be reminded of the delay. Mice then had 10 free choice trials. The side with the large reward was matched to the chained side training. If mice responded for the small reward, an ITI occurred based on the delay for that session. If mice chose the large reward, the center hole blinked for the duration of the delay. On the Worsening schedule, mice experienced 12 trials of increasing delays – 0 s, 4 s, 12 s, 20 s, 28 s delays – within one session. The Improving schedule was similar but in the reverse orientation – 28 s, 20 s, 12 s, 4 s, 0 s delays – within one session. Mice could only move on to the next delay if they responded on all 12 trials for that delay and collected the reward. In order to track progression of delay discounting stability, we have included subplots with fewer days according to the start and end of training (day matched with the last round of training and probability discounting; [33]).

#### Worsening and Improving Probability Discounting.

Animals transitioned directly from the last delay discounting schedule to the first probability discounting schedule because the structure of the task (location of large and small reward, response order) did not change, only the rule governing payout of the large reward. Mice underwent 10 days of Worsening Probability Discounting I and followed by 8 days of Improving Probability Discounting I, 9 days of Worsening Probability Discounting II and 9 days of Improving Probability Discounting II, and 16 days of Worsening Probability Discounting III and 18 days of Improving Probability Discounting III. As in delay discounting, the same side resulted in a large reward or small reward. Both rewards were immediate, but the large reward was delivered probabilistically. All probability blocks consisted of free choice trials. On the Worsening schedule, mice experienced 20 trials of decreasing probability of reward delivery – 100%, 75%, 50%, 25%, 12.5% chance of reward – within one session. The Improving schedule was similar but in the reverse orientation – 12.5%, 25%, 50%, 75%, 100% probabilities – within one day. If the trial was not rewarded, a feedback house light would blink indicating a non-rewarded trial. We used the house light instead of the screen in order to help mice distinguish between discounting tasks (i.e. delay discounting used screen to signal delay, probability discounting used house light to signal reward loss). Mice could only move on to the next probability if they responded to all 20 trials for that probability and collected the reward. In order to track progression of probability discounting stability, we have included subplots with fewer days according to the start and end of training (day matched with the last round of training and delay discounting; [33]).

### 2.4. Computational modeling

To quantitatively examine how the value of rewards vary as a function of delay, we fit an exponential discounting model [40], shown as in the equation below:

$$V=Ae^{KD},$$

where *V* is the subjective or discounted value of the delayed reward, *A* is the amount or magnitude of the delayed reward, and *D* is the length of delay. *k* is a free parameter that reflects the discounting rate: the larger *k* is, the steeper the discounting of reward value; the smaller *k* is, the slower the discounting of reward value. *k* is determined by the fit of the model to the actual data.

Then, we fit a similar exponential model [44] to examine how uncertainty of reward affects the value of reward.


$$V=Ae^{-h\left(\frac{1}{p}-1\right)}$$

In this model, *p* is the probability of reward and *A* is the magnitude of the reward. The free parameter *h* dictates how steep the change in the value of reward is as a function of the reward probability. Thus, *h* in the probability discounting model and *k* in the above delay discounting model both describe how rapidly the value of a reward is discounted, either by uncertainty or delay of the reward.

For both models, the action selection was performed based on a Softmax probability distribution:

$$P(A)=\frac{e^{V_{A}*\beta }}{\sum_{j}e^{V_{j}*\beta }},$$

Where V_A_ corresponds to the subjective reward value of action A, and a second free parameter inverse temperature β determines the level of decision noise. When inverse temperature is high, the decision noise is low, which means more exploitation of the action with high subjective value; when inverse temperature is low, the decision noise is large, which means more random exploration regardless of value. The optimized parameters were obtained through minimization of the negative log likelihood of the models.

### 2.5. Statistical analysis

Delay discounting and probability discounting data were analyzed using R Studio using the lme4 package. Linear mixed models were fit to preference proportion data for both delay discounting and probability discounting with fixed effects of sex (males and females), schedule (Improving and Worsening), delay/probability and random effect for subjects (Figs. 2 & 3). Win-stay scores were calculated by taking the number of times animals stayed on the large risky side after receiving Ensure on the previous trial divided by the total number of times animals received Ensure on the large side. Lose-shift scores were calculated by taking the number of times animals did not receive Ensure on the large side and switched to the small certain side divided by the total number of times animals did not receive rewards on the large side (regardless if they shifted or not). These scores were then analyzed using linear mixed models with fixed factors of sex and schedule and random effect for subjects (Fig. 4). Computational modeling results were fit to discounting and inverse temperature parameters (Fig. 5) and were analyzed with linear-mixed models with task and schedule as the fixed factors and random effects for subjects.

Response time for small and large choice data were fit with the same factors (Figs. 6 & 7). Response times greater than 3 standard deviations away from the mean were eliminated from analysis. Beam break data were fit with fixed factors of sex, schedule, and break location (front and back) and random effect for subjects (Figs. 8 & 9). 2 mice (1 male, 1 female) were excluded from beam break analysis because of a mal-function in infrared beams. Beam breaks greater than 3 standard deviations aways from the mean were eliminated. In cases where sex was not significant based on a log-likelihood ratio test, sex was removed from the linear mixed models. Data were fit for all days in the main plots and for days 1–3 and days 6–8 in the subplots in order to track the progression of discounting stability. For all statistics, an alpha of 0.05 was used.

## 3. Results

**Fig. 1.**
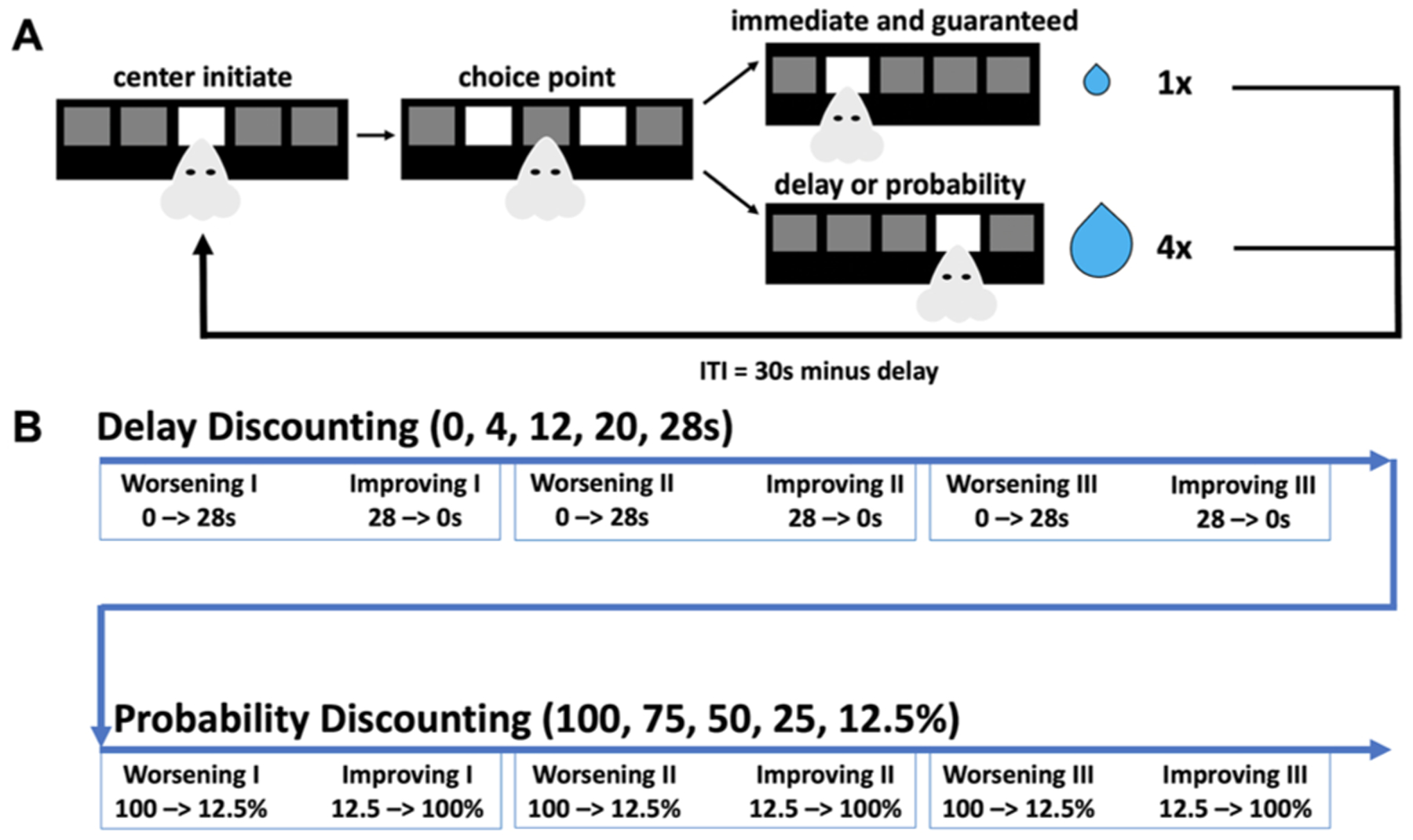
Schematic of delay discounting and probability discounting. **A.** Mice responded for large and small rewards after initiating the trial by poking the center hole. Mice then chose one side for a small immediate reward (6.25 μl) or a large delayed/probabilistic reward (25 μl). Small choices on the delay schedule were followed by an ITI to time match the delay of the large reward. **B.** Mice began discounting tasks on the Worsening schedule (i.e. increasing delay) for 10 days and then were switched to the Improving schedule (i.e. decreasing delay) for 9 days. Schedules continued to switch two more times. Mice then experienced probability discounting on the Worsening schedule (i.e. increasing uncertainty) for 10 days and then were switched to the Improving schedule (i.e. decreasing uncertainty) for 8 days. Schedules again switched two more times.

Age-matched male and female wildtype mice (n = 15, 8 males, 7 females, strain B6129SF1/J) were trained to perform sequential discounting tasks using touchscreen operant chambers (Fig. 1A). This permitted us to test the extent to which choice preferences and discounting were “anchored” by the initial delay/probability of the large reward. Trials were paced to require 30 s minus the length of the delay before the next trial could be initiated to remove the ability to complete all trials more quickly [43] that may contribute to prior demonstrations of greater action impulsivity. Repeated sessions of delay and probability discounting allowed us to study anchoring effects and choice strategy, as well as ask questions about sex differences and individual differences in preferences for delay and probability simultaneously.

### 3.1. Delay discounting

#### Anchoring effects to delayed rewards are reduced with experience.

Rodent models of delay discounting have previously used latin square design of delays [35], but structured delay schedules have not widely been used in mice (e.g. ascending or descending schedules). Because of the novelty of these schedules, we initially put mice through delay discounting in order to test whether sex is an important factor in anchoring effects induced by shifting schedules (i.e. “Worsening” and “Improving”). Research suggests uncertainty in discounting tasks can produce sex-specific effects [23], thus we wanted to test whether sex was important in anchoring responses to Worsening and Improving schedules. Here, we present the data from each round of Worsening and Improving schedules grouped together.

**Fig. 2.**
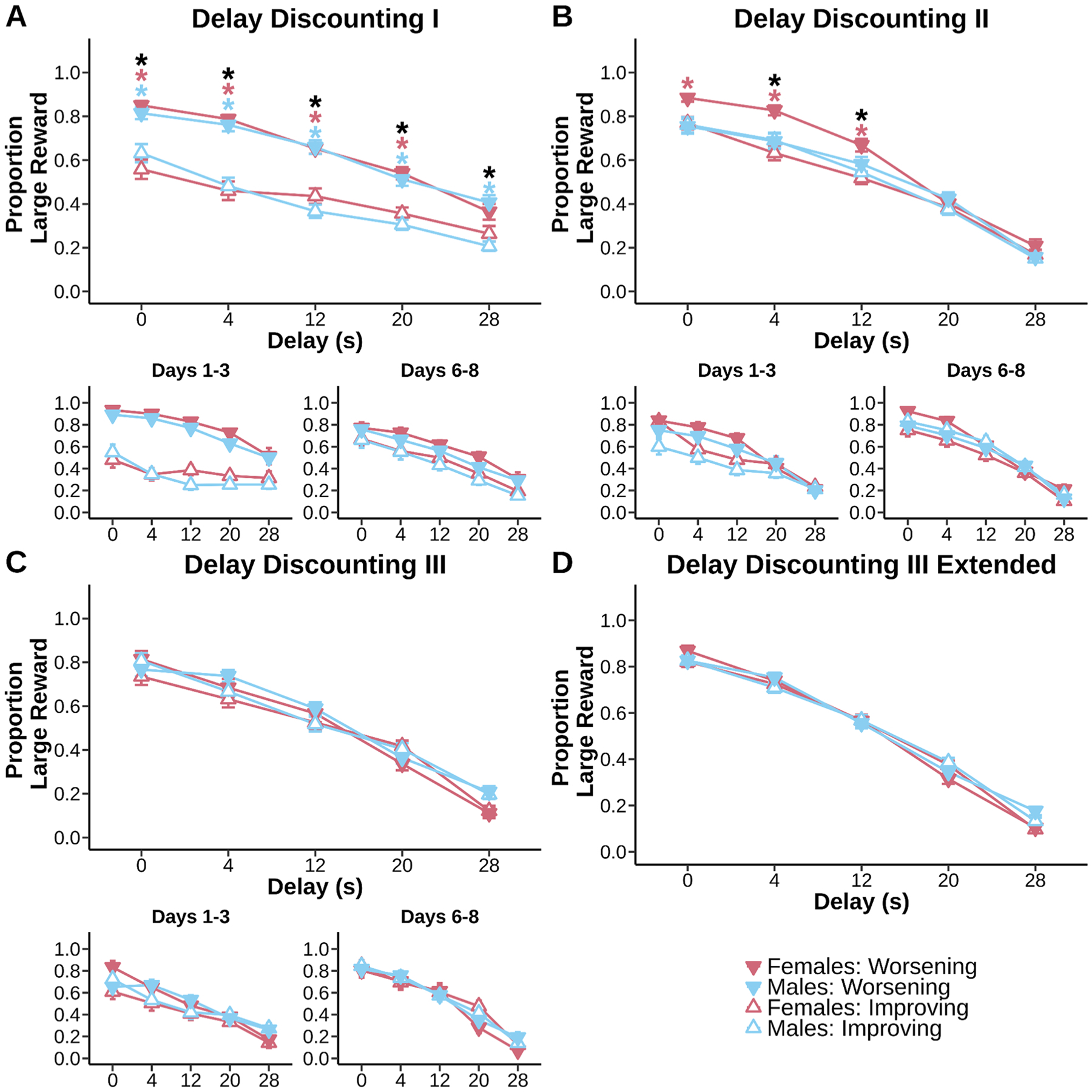
Anchoring effects in delay discounting are eliminated with extended experience. Main plots represent a summary of all days of training. Subplots represent day matched periods of training. **A:** Mice of both sexes responded less for the large reward at the longest delays compared to the shortest, even with minimal experience on the tasks. However, there was a significant anchoring effect, such that mice in the Improving (started at 28 s delay) condition had a persistent reduction in choosing the large reward compared to the Worsening (started at 0 s delay) condition. Anchoring was persistent from the beginning to the last days of training as indicated by schedule × delay effects (Worsening > Improving: 0 s, p < 0.001; 4 s, p < 0.001; 12 s, p < 0.001; 20 s, p < 0.001; 28 s, p = 0.0020). **B:** Anchoring effects lingered into the second session of delay discounting (Worsening > Improving: 4 s, p = 0.0021; 12 s, p = 0.0025), but were most pronounced in females. Anchoring became more apparent during the last days of training in females. **C&D:** Data in Fig. C represent the first 8 days, data in Fig. D represent all days of training. With continued experience, females and males reached similar discounting rates and no longer anchored their preference according to whether sessions started with a long or short delay. Anchoring was present early into training but disappeared with continued experience. Figures depict mean ± SEM, black asterisks indicate significant anchoring effects (schedule × delay interactions) while colored asterisks indicate significant sex effects (planned post hoc comparisons of Worsening and Improving schedules within a sex at each timepoint) of p < 0.05.

The first time mice experienced both the Worsening and Improving delay discounting schedules (Delay Discounting I), their preferences for the large reward across the entire sessions were heavily anchored by the initial delay experienced (Fig. 2A, main effect of schedule, F_(1, 15.01)_ = 55.26, p < 0.001). Mice learned to shift their choice from the delayed side to the immediate side as delay increased (Fig. 2A, main effect of delay, F_(4, 60.17)_ = 52.26, p < 0.001). Evidence suggested mice exhibited delay specific sensitivity according to schedule (Fig. 2A, schedule × delay interaction, F_(4, 1081.77)_ = 5.84, p < 0.001). Male mice had a large choice preference at all delays on the Worsening schedule compared to the Improving schedule (0 s: p = 0.0316; 4 s: p < 0.001; 12 s: p < 0.001; 20 s: p = 0.0129; 28 s: p = 0.0170). Female mice had a large choice preference on the Worsening schedule compared to the Improving schedule except for at the 28 s delay (0 s: p < 0.001; 4 s: p < 0.001; 12 s: p = 0.0138; 20 s: p = 0.0406). Mice exhibited this anchoring effect both at the beginning of training (Fig. 2A, days 1–3: main effect of schedule, p < 0.001) and end of training (Fig. 2A, days 6–8: main effect of schedule, p = 0.0136). Although increased large choice preference across delays of the Worsening schedule was specific to the beginning of training (Fig. 2A, days 1–3: schedule × delay interaction, p < 0.001). These results indicate temporal uncertainty induced by a schedule shift immediately caused anchoring effects. Preference for the large reward at all delays was greater when it was “anchored” by an initial 0 s delay than an initial 28 s delay.

Despite a second round of testing (Fig. 2B, Delay Discounting II), anchoring effects persisted (Fig. 2B main effect of schedule, F_(1, 15)_ = 6.81, p = 0.0197). Animals continued to show strong discounting to each transition of delay (Fig. 2B, main effect of delay, F_(4, 60.21)_ = 116.42, p < 0.001). Mice adjusted choice across delays according to schedule (Fig. 2B, schedule × delay interaction, F_(4, 1251.92)_ = 2.98, p = 0.018). Anchoring was especially apparent in female mice (Fig. 2B, sex × schedule × delay interaction, F_(4, 1251.92)_ = 5.21, p < 0.001). Anchoring in female mice was specific to the smallest delays (0 s: p = 0.0465; 4 s: p < 0.001; 12 s: p = 0.0057). Mice retained anchoring early into training (Fig. 2B, days 1–3: main effect of schedule, p = 0.0123), but the effect was reduced with additional training (Fig. 2B, days 6–8: main effect of schedule, p = 0.207). However evidence suggests female mice retained sensitivity to differences in schedule order with experience (Fig. 2B, days 6–8: sex × schedule interaction, p = 0.0238; days 6–8: sex × schedule × delay interaction ns, p = 0.0577). These data suggest that female mice have an increased sensitivity to anchoring effects and/or increased sensitivity to unexpected changes in the task rules.

By the time animals were tested on Delay Discounting III (Fig. 2C), there were no longer any anchoring effects or differences apparent in choice (main effect of schedule ns, p > 0.05). Animals showed robust discounting to each delay (Fig. 2D, main effect of delay, F_(4, 60)_ = 175.55, p < 0.001) that had some suggestion of delay specific effects between schedules (Fig. 2D, schedule × delay interaction, F_(4, 2355)_ = 5.79, p < 0.001) but did not reveal any post hoc effects. Mice expressed anchoring effects early into training still (Fig. 2C, days 1–3: main effect of schedule, p = 0.0282) of which was more apparent in females (Fig. 2C, days 1–3: sex × schedule × delay interaction, p = 0.0140). Increased training mitigated anchoring effects (Fig. 2C, days 6–8: main effect of schedule ns, p > 0.05; days 6–8: schedule × delay interaction, p = 0.0064). Taken together, our results suggest mice have the ability to form similar choice preferences across two different schedule orientations. Anchoring effects are eliminated by the end of discounting for both male and female mice. However, females exhibited persistent anchoring until the penultimate round of discounting.

### 3.2. Probability discounting

#### Anchoring effects are persistent when discounting risky rewards.

We put mice through probability discounting with “Worsening” and “Improving” schedules in order to challenge anchoring in response to uncertain large rewards. We tested mice on these schedules to see if risky rewards produced anchoring effects in a similar manner. If males and females are differently affected by uncertainty, it would stand to reason that those differences may be most reflected in the anchoring effects. We present the data from each round of Worsening and Improving schedules grouped together.

**Fig. 3.**
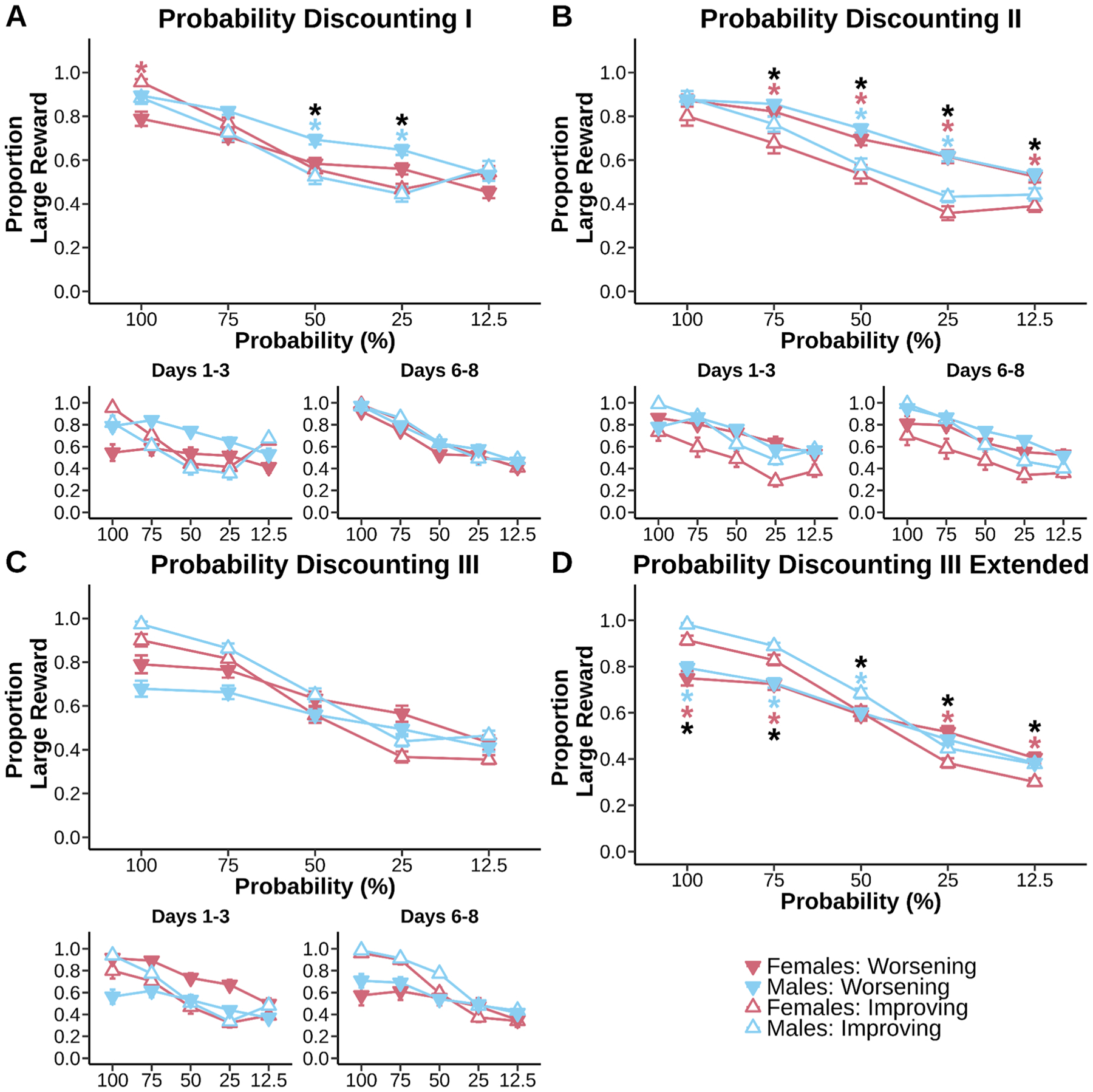
Anchoring effects continue to influence probability discounting behavior after extended experience. Main plots represent a summary of all days of training. Subplots represent day matched periods of training. **A:** Regardless of uncertainty orientation, males and females choose the large reward less often with increasing risk. Interaction effects of schedule × probability (Worsening > Improving) were specific to the 25% (p = 0.001) and 50% (p = 0.0210) probabilities. **B:** For the second round of probability discounting, mice showed significant discounting of rewards and evidence of anchoring. Interaction effects of schedule × probability (Worsening > Improving) were specific to the 12.5% (p < 0.001), 25% (p < 0.001), 50% (p < 0.001), and 75% (p < 0.001) probabilities. Females were sensitive to uncertainty throughout the session (Worsening > Improving) whereas males were only sensitive at the 50% and 25% probabilities (Worsening > Improving). **C&D:** Data in Fig. C represent the first 8 days, data in Fig. D represent all days of training. With extended training, mice were sensitive to schedule effects at all probabilities where large choice preference was greater on the Worsening schedule than the Improving schedule at probabilities below 50% chance (12.5%: p = 0.0230, 25%: p < 0.001), but switched to a greater large choice preference on the Improving schedule compared to the Worsening schedule when probabilities were at or above 50% chance (50%: p = 0.0410, 75%: p < 0.001%, and 100%: p < 0.001). Females showed decreased risky choice preference on the Improving schedule at risky probabilities, but increased risky choice preference on the Improving schedule at safe probabilities. Male mice made schedule specific adjustments only at safer probabilities (50% probability and higher). Figures depict mean ± SEM, black asterisks indicate significant anchoring effects (schedule × probability interactions) while colored asterisks indicate significant differences within a sex in responding to specific probabilities (planned post hoc comparisons) of p < 0.05.

Unlike delay discounting, the first time mice experienced both the Worsening and Improving probability discounting schedules (Probability Discounting I), their preferences for the large reward across the entire sessions were not heavily anchored overall by the initial probability experienced (Fig. 3A, no main effect of schedule, F_(1, 15.01)_ = 0.42, p = 0.527). All mice showed significant discounting on both schedules, as measured by changes in their preferences for the large reward (Fig. 3A, main effect of probability, F_(4, 60.88)_ = 123.43, p < 0.001). However, mice showed schedule specific preferences depending on the probability (Fig. 3A, schedule × probability interaction, F_(4, 1122.52)_ = 21.15, p < 0.001) and differences in the magnitude of preference between schedules (Fig. 3A, sex × schedule interaction, F_(1, 15.01)_ = 4.83, p = 0.0441). Males chose the large reward more often on the Worsening schedule compared to the Improving schedule when the chance of winning risky rewards was at 25% (p = 0.0016) and 50% (p = 0.0102). Females increased large choice preference on the Worsening schedule compared to the Improving schedule when large rewards were guaranteed (100% chance, p = 0.0121). Anchoring did not appear throughout training (Fig. 3A, days 1–3 and days 6–8, main effect of schedule ns, p > 0.05). However, differences in preference at different probabilities between schedules were present at the start of training (Fig. 3A, days 1–3, schedule × probability interaction, p < 0.001) and the end of training (Fig. 3A, days 6–8, schedule × probability interaction, p = 0.0144). Male specific differences in preference seemed to appear early on (Fig. 3A, days 1–3, sex × schedule × probability interaction ns, p = 0.0542). These data indicate mice were not as affected by schedule effects compared to delay discounting, especially with greater experience.

As mice continued to gain more experience with the task design during the second round of testing (Fig. 3B, Probability Discounting II), anchoring effects became more apparent (Fig. 3B, main effect of schedule, F_(1, 15)_ = 37.74, p < 0.001). All mice continued to show strong discounting to each transition of probability (Fig. 3B, main effect of probability, F_(4, 60)_ = 128.56, p < 0.001). These anchoring effects were specific to different probabilities of risky rewards (Fig. 3B, schedule × probability interaction, F_(4, 1140)_ = 9.08, p < 0.001). Female mice significantly reduced their large choice preference on the Improving schedule compared to the Worsening schedule when rewards were risky (12.5% chance, p = 0.0149; 25% chance, p < 0.001; 50% chance, p = 0.0025; 75% chance, p = 0.0082). Male mice started to show reduced large choice preference on the Improving schedule compared to the Worsening schedule at 25% chance of reward (p < 0.001) and 50% chance of reward (p < 0.001). While the overall data suggests no sex effects, male and female mice did have a difference in large reward preference early into training (Fig. 3B, days 1–3: main effect of sex, p = 0.0163). Anchoring effects were present early into training (Fig. 3B, days 1–3: main effect of schedule, p < 0.001) and at the end of training (Fig. 3B, days 6–8: main effect of schedule, p = 0.0013). Early training emphasized a difference in large preference at different probabilities between schedules (Fig. 3B, days 1–3, schedule × probability interaction, p < 0.001) and differences in overall preference between schedules (Fig. 3B, days 1–3, sex × schedule interaction, p < 0.001). Mice shifted to differences in large preference based on specific probabilities rather than generally between the schedules (Fig. 3B, days 6–8, sex × probability interaction, p = 0.0188). These data indicate anchoring effects reappeared after mice gained more experience where reward receipt was probabilistic.

By the time animals were tested on Probability Discounting III (Fig. 3C), anchoring effects were still pervasive (Fig. 3D, main effect of schedule, F_(1, 14.22)_ = 7.80, p = 0.0142). All mice showed significant discounting (Fig. 3D, main effect of probability, F_(4, 60.57)_ = 315.46, p < 0.001). Mice again showed schedule specific preferences depending on the probability (Fig. 3D, schedule × probability interaction, F_(4, 2198.20)_ = 45.12, p < 0.001) and within sex (Fig. 3D, sex × schedule interaction, F_(1, 14.22)_ = 4.98, p = 0.0422). Female mice reduced risky choices on the Improving schedule compared to the Worsening schedule when uncertainty was high (12.5% chance, p = 0.0106; 25% chance, p < 0.001) and increased risky choices on the Improving schedule compared to the Worsening schedule when uncertainty was low (75% chance, p = 0.0102; 100% chance, p < 0.001). Male mice also made risk-based adjustments but only to maximize rewards on the Improving schedule compared to the Worsening schedule when uncertainty was at or above chance level (50% chance, p = 0.0350; 75% chance, p < 0.001; 100% chance, p < 0.001). Mice showed a reduction of anchoring early into training, but anchoring resurfaced with additional exposure to probabilistic rewards (Fig. 3C, days 6–8: main effect of schedule, p < 0.001). Large reward preference remained sensitive to the schedule type and specific probability experienced (Fig. 3C, days 1–3: schedule × probability interaction, p < 0.001; days 6–8: schedule × probability interaction, p < 0.001). Schedule directed large reward preference was present early into training (Fig. 3C, days 1–3: sex × schedule interaction, p < 0.001) but not late into training. These results demonstrate that females and males made schedule specific adjustments in avoiding losses around an immediately risky schedule (i.e. Improving probability discounting) and that mice continued to remain sensitive to schedule effects with extended training.

### 3.3. Win-stay/lose-shift adaptations to risk order

#### Probability discounting schedules that decrease trial block uncertainty promote win-stay choices.

Win-stay/lose-shift behaviors are important indicators of strategy specific adaptations to wins and losses. Win-stay ratios were calculated by dividing how often mice stayed on the same risky side after being rewarded divided by all rewarded risky responses. Lose-shift ratios were calculated by dividing how often mice switched to the small guaranteed side after not receiving a large risky reward divided by all losses on the risky side. We wanted to study if females and males made specific adaptations to wins and losses in response to different risk orientations, which can be a source of choice differences [55].

**Fig. 4.**
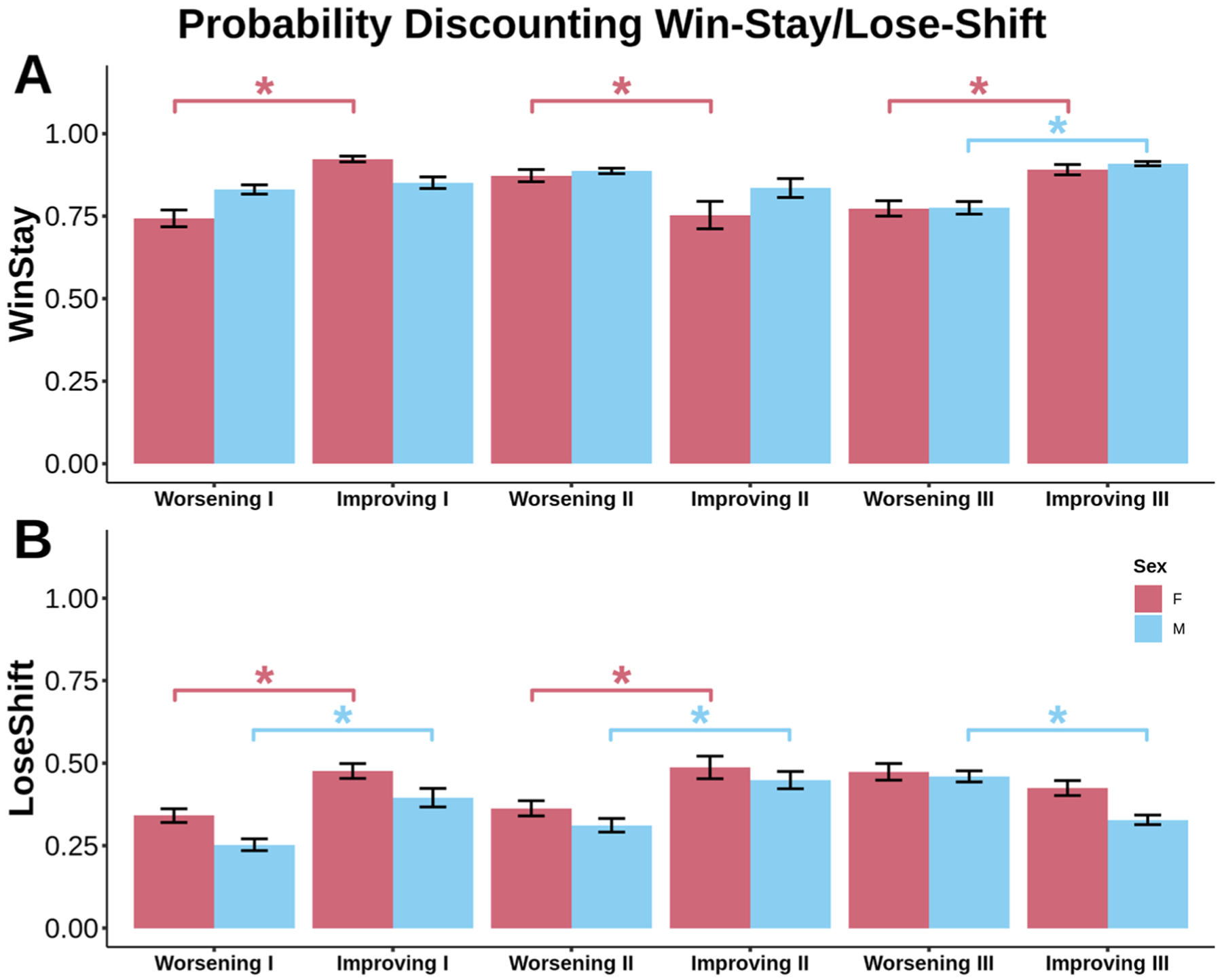
Adaptations to wins and losses with experience. We examined whether tendencies to win-stay/lose-shift explained sex differences in probabilistic responding. Win-stay ratios were calculated by dividing how often mice stayed on the same risky side after being rewarded divided by all rewarded risky responses. Lose-shift ratios were calculated by dividing how often mice switched to the small guaranteed side after not receiving a large risky reward divided by all losses on the risky side. **A:** Female mice specifically learned to increase win-stay behavior after their first experience with risky rewards. Females continuously make adjustments to win-stay behavior throughout their exposure to both Worsening and Improving schedules. By the end of probability discounting, males and females learned to make more win-stay choices on the Improving schedule. **B:** Male and female mice adapted to risky rewards by increasing their lose-shift behavior on an initially risky schedule (i.e. Improving). By the end of probability discounting, only males showed lose-shift specific adaptations. Figures depict mean ± SEM, colored asterisks indicate significant within sex schedule effects (planned post hoc comparisons) of p < 0.05.

At the beginning of probability discounting, mice increased win-stay behavior on the Improving schedule (Fig. 4A, main effect of schedule, F_(1, 255)_ = 34.38, p < 0.001), but a sex × schedule interaction (Fig. 4A, F_(1, 255)_ = 21.77, p < 0.001) indicates only females increased win-stay behavior on the Improving schedule (p < 0.001). Mice also increased lose-shift behavior on the Improving schedule (Fig. 4B, main effect of schedule, F_(1, 255)_ = 76.87, p < 0.001). Both male (p < 0.001) and female (p < 0.001) mice exhibited increased lose-shift behavior on the Improving condition. These results suggest both male and female mice adjust their behavior to increased initial uncertainty in a similar way, but females specifically showed a schedule specific distinction in maximizing rewards.

Mice continued to make win-stay choice adaptations throughout the second round of probability discounting (Fig. 4A, main effect schedule, F_(1, 270)_ = 10.50 p = 0.0013) which was again specific to female mice (Worsening II > Improving II, p = 0.0116) and continued to make loss specific adaptations (Fig. 4B, main effect of schedule, F_(1, 255)_ = 32.50, p < 0.001). Increased lose-shift behavior on the Improving schedule was found again in both males (p < 0.001) and females (p = 0.0016). Female mice again differentiated themselves from male mice because females shape their choice behavior around adjustments in win-stay behavior.

As mice finished probability discounting, male (p < 0.001) and female (p < 0.001) mice continued to increase win-stay behavior on the Improving schedule (Fig. 4A, main effect of schedule, F_(1, 460.28)_ = 55.94, p < 0.001). A main effect of schedule was found for lose-shift behavior (Fig. 4B, F_(1, 461.30)_ = 26.65, p < 0.001), but only male mice exhibited decreased aversion to losses (Fig. 4B, F_(1, 461.30)_ = 5.16, p = 0.0235) on the Improving schedule (p = 0.0024). Females made schedule specific adaptations to wins even with minimal experience whereas males required extended training to show win-stay schedule effects. Males and females were equally loss avoidant for the most of training, extended training reduced schedule specific effects in females whereas males remained sensitive to losses.

### 3.4. Computational models of choice variability in delay and probability discounting

#### Decision noise is a major contributor to delay and probability discounting.

Win-stay/lose-shift analyses revealed some learning specific effects, but did not explain changes in preference over renditions of the task. We posited that snapshot win-stay/lose-shift analyses might not capture broader trends driving choice behavior. There is a growing amount of evidence suggesting exploration is a key latent variable driving choice behavior [14,15]. Therefore, we pursued two discounting models for delay and probability, each incorporating a value parameter (*k* for delay, *h* for probability; [22]) and an inverse temperature parameter capturing variability in choice around these value preferences (β). This allowed us to track adaptations in value and choice within and between tasks. We excluded sex as a factor to increase power and better detect schedule specific adaptations. Data were fit to an exponential model rather than a hyperbolic model for delay and probability discounting because those provided better fits according to AIC (Delay Discounting Hyperbolic AIC (Improving = 31,055.57 & Worsening = 29,689.31) > Delay Discounting Exponential AIC (Improving = 29,910.06 & Worsening = 28,364.77); Probability Discounting Hyperbolic AIC (Improving = 52,026.82 & Worsening = 54,768.72) > Probability Discounting Exponential AIC (Improving = 47,774.92 & Worsening = 52,824.56)).

**Fig. 5.**
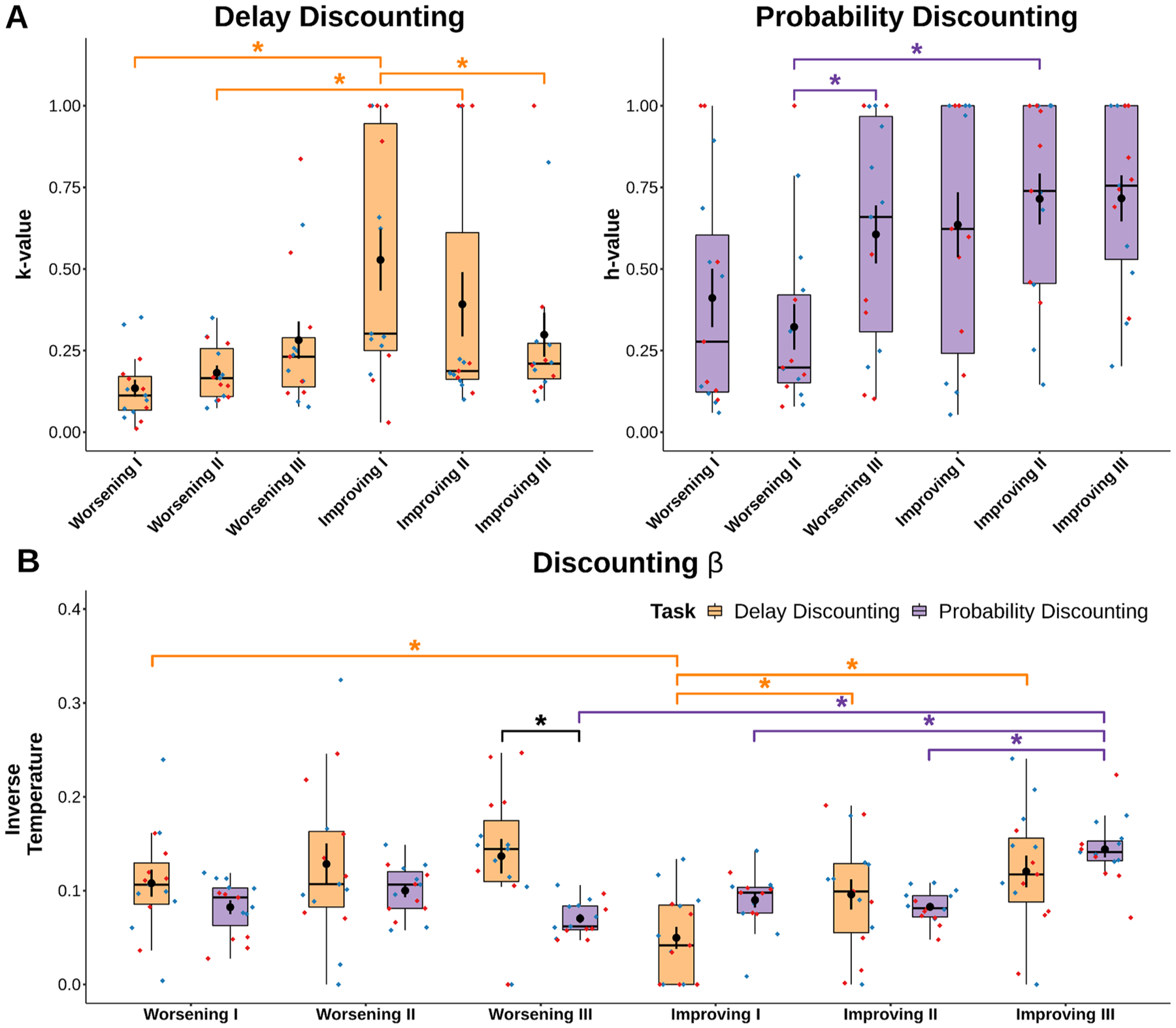
Discounting as explained by value and decision noise. We modeled discounting rates (*k* and *h*) and decision noise (inverse temperature, β) for each mouse across all days of testing. **A:** Mice alter *k* as they continuously adapt to differences in delay order. Mice quickly converge on a similar *h* value despite changes in probability order. **B:** Inverse temperature (β, reflecting decision noise) parameters for delay and probability discounting reveal large changes in the noisiness of choices as schedules change. Inverse temperature increased over time with extended experience with both tasks (especially on Improving schedules), indicating decreased decision noise in choices with experience. Figures depict individual points for males (blue) and females (red) and box-and-whisker plots with mean ± SEM overlaid; asterisks indicate significance of p < 0.05.

Analysis of the delay discounting rate parameter (*k*) revealed mice had smaller discounting rates for the Worsening condition (Fig. 5A, main effect of schedule, F_(5, 75)_ = 8.53, p < 0.001). Mice had a steeper discounting rate initially on the Improving condition compared to the Worsening condition (Fig. 5A, p < 0.001), possibly due to uncertainty induced by a switch in orientation. Mice were steeper still for the second round of Improving and Worsening schedules (Fig. 5A, p = 0.0371). Mice became more willing to endure delays for large rewards between the first and third round of Improving schedules (Fig. 5A, p = 0.0171). Probability discounting rates (*h*) were also schedule dependent (Fig. 5A, F_(5, 75)_ = 8.12, p < 0.001), especially for the second session of discounting (Fig. 5A, Improving II > Worsening II, p < 0.001). Mice became steeper between for the third round of Worsening discounting compared to the second (Fig. 5A, p = 0.0104). Mice became more cost enduring with training for the large reward in delay discounting, but were generally cost avoidant for the large reward in probability discounting.

Between-task analyses of the inverse temperature in our discounting model revealed delay and probability discounting noise were similar in magnitude (Fig. 5B, main effect of task ns, p > 0.05). However there were specific task × schedule interactions which were present (Fig. 5B, F_(5, 75)_ = 8.37, p < 0.001). Mice engaged in increased repetitive choice on Worsening III of delay discounting compared to probability discounting (Fig. 5B, p < 0.001).

Within-task comparisons of the choice parameter revealed mice adapt to initial temporal uncertainty caused by an orientation switch through increased sampling of reward options (Fig. 5B, Worsening I > Improving I, p = 0.0021). Mice learned to decrease decision noise throughout their experience with Improving delay discounting (Fig. 5B, Improving I < Improving II, p = 0.0430; Improving I < Improving III, p < 0.001). Mice similarly learned to decrease decision noise with Improving probability discounting (Fig. 5B, Improving I < Improving III, p = 0.0065; Improving II < Improving III, p < 0.001). By the end of training, decision noise was greater for Worsening probability discounting compared to Improving probability discounting (Fig. 5B, Improving III > Worsening III, p < 0.001).

Mice choice preference in delay discounting demonstrates that differences in behavior can at least in part be explained by changes in *k*, the parameter describing the discounted value of the large reward. That is, when we model the behavior of these animals, the best fit model adjusts the *k* value over the progression of Delay Discounting (I, II, III), meaning that differences in animals’ choices across the progression of delay discounting tasks reflect in part true changes in the discounted value of the large reward. When animals’ choice curves shift, this reflects changes in how much they value the reward, not only changes in how variable or noisy they are in responding. In contrast, in the probability discounting task, the comparable *h* value, the discounted value of the large reward, is stable - meaning that changes in behavior in this task are not described well by models adjusting the value of the reward. Rather, adjustments in the inverse temperature parameter, which measures how noisy animals are in adhering to their values, provide the best fit - suggesting that exploratory noise is a major driver in decision making in the probability task.

### 3.5. Choice response times during delay and probability tasks

#### Examination of response times and locomotion as indexes of cognitive uncertainty and changing task engagement, respectively.

Exploration has a number of different meanings in different fields. Our computational model contains an inverse temperature parameter that reflects the “noisiness” of adhering to a value based decision rule, and this noisiness could emerge from one of several sources. First, it could reflect cognitive uncertainty and exploration processes; these can be reflected in increasing choice response times as decisions become more costly or more uncertain [17,34,52]. Alternatively, noisiness in behavior could reflect changing engagement with the tasks over time, which can be reflected in the locomotor behavior or unobserved behaviors of animals as they complete the tasks [36,58]. As the exploration in both of our tasks goes down with experience, we should see changes in *choice response times* with task experience if this exploration reflects aspects of cognitive uncertainty, versus changes in *locomotor behavior* if exploration reflects task disengagement. We addressed each of these possibilities by examining choice response times and locomotor behavior incidentally captured in the chambers during collection of the above task performance data.

#### 3.5.1. Choice response times track temporal uncertainty only in animals experienced with delay discounting

Choice response times are subject to change according to schedule orientation in discounting tasks [45]. Research suggests discounting choice response times increase when the subjective value of the large delayed option equals the delay-free option [4,45]. These researchers suggested reaction time reflects conflict of equally valued options, something that should reveal itself with increasing or decreasing costs. Response times then could reflect the difficulty present in our within-subject tasks where choice preference is anchored to what is learned in the preceding schedule. If differences in exploration over training on the delayed discounting task do reflect increasing conflict as animals better understand the temporal cost of a choice, then we should see the emergence of delay-dependent increases in choice response times over our training schedules, particularly as anchoring effects go away. Indeed, we found that choice response times tracked the degree of temporal uncertainty of each delay in the most highly trained sections of the task (Delay Discounting III) compared to naive performance of the task (Delay Discounting I).

**Fig. 6.**
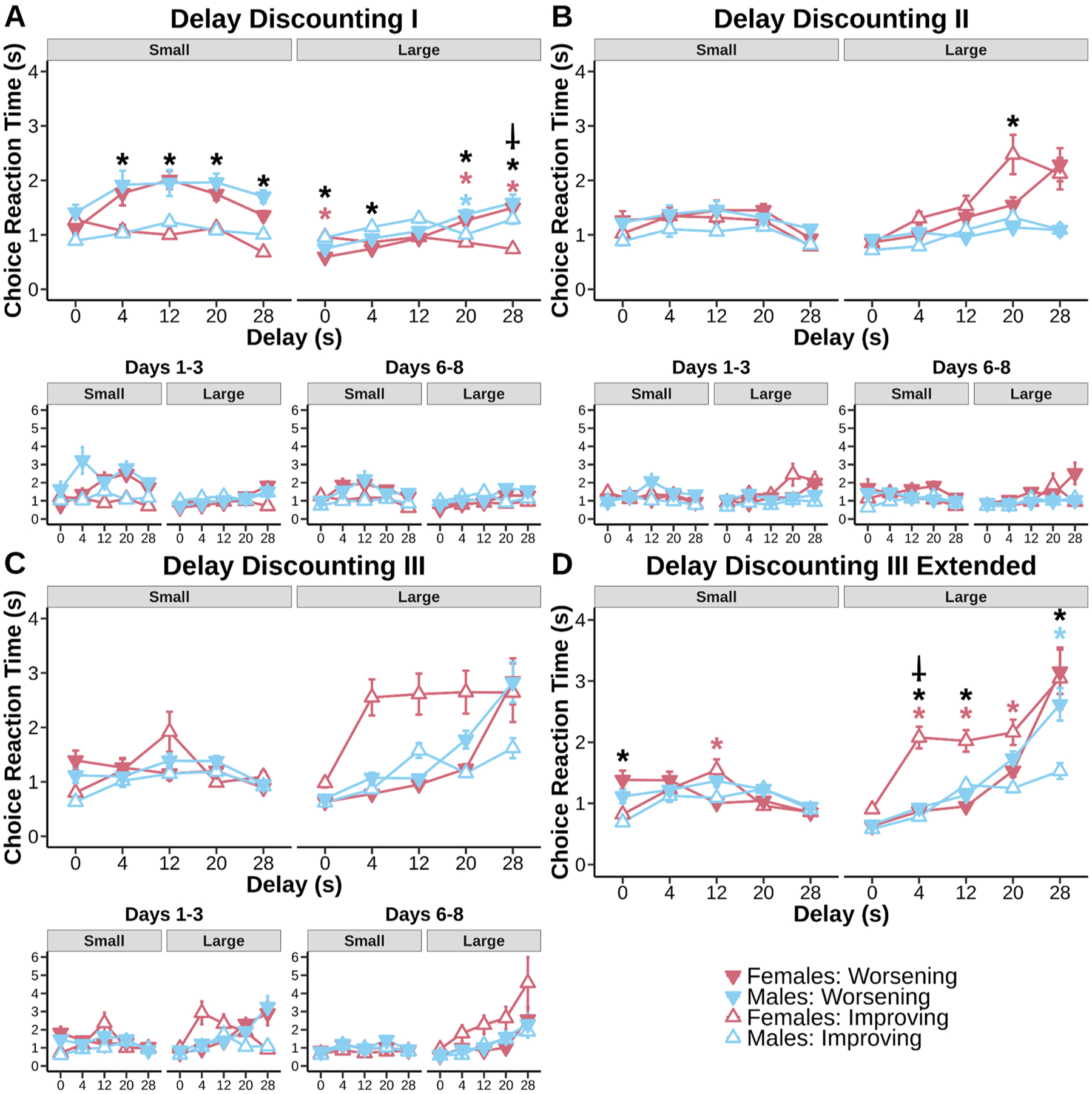
Response times become more influenced by delay cost on a choice as animals gain experience with delay discounting. Main plots represent a summary of all days of training. Subplots represent day matched periods of training. **A:** Mice made slightly slower choice responses for both large and small rewards when the delays were Worsening relative to when they were Improving. **B:** Mice converged choice response times for small and large choice response times for Delay Discounting II. The clearest delay effect was noticeable at the 20 s delay. **C&D:** Data in Fig. C represent the first 8 days, data in Fig. D represent all days of training. Extended experience with delay discounting induced schedule effects in small and large choice response times. All mice were slower on the Improving schedule 0 s delay, but only female mice were slower on the Improving schedule at the 12 s delay. Females slowed down for large choices when the schedule was Improving for the 4 s, 12 s, and 20 s delays. Only males slowed down at the largest delay on the Worsening schedule. Figures depict mean ± SEM. Black asterisks indicate significant schedule effects (schedule × delay interactions), black daggers pointing up indicate between sex effects on the Improving schedule (sex × schedule × delay interactions) of p < 0.05. Colored asterisks indicate significant schedule effects within a sex (sex × schedule × delay interactions) of p < 0.05.

In order to demonstrate this, we took the time from choice presentation to decision made as an index of choice response time. These data are depicted in Fig. 6. The first round of delay discounting showed immediate evidence of the effect of delay on response time. Anchoring has an effect on choice response times where expectation of an initial delay-free period (i.e. from the Worsening schedule) causes mice to speed up on the Improving schedule. It is likely that small choice response times in this case reflect the learning effect of experiencing delay for the first time. Both the small immediate option (Fig. 6A, main effect of delay, F_(4, 57.42)_ = 4.29, p = 0.0042) and large delayed option (Fig. 6A, main effect of delay, F_(4, 40.32)_ = 33.41, p < 0.001) showed evidence of increased response time with delay. Small choice response times showed evidence of anchoring (Fig. 6A, main effect of schedule, F_(1, 14.93)_ = 45.06, p < 0.001). Small choice schedule differences in delay (Fig. 6A, schedule × delay interaction, F_(4, 5460.37)_ = 4.82 p < 0.001) were apparent as soon as rewards were delayed in which mice were faster to respond for small rewards on the Improving schedule compared to the Worsening schedule (4 s: p < 0.001; 12 s: p < 0.001; 20 s: p < 0.001; 28 s: p < 0.001). Large choice response times also showed evidence of anchoring (Fig. 6A, main effect of schedule, F_(1, 14.85)_ = 4.91, p = 0.0427). Large choice schedule effects per delay (Fig. 6A, schedule × delay interaction, F_(4, 5603.65)_ = 26.99, p < 0.001) were found across most delays. Mice generally were slower at smaller delays when the schedule was decreasing in delay (Improving > Worsening, 0 s: p < 0.001; 4 s: p = 0.0393), but sped up at larger delays (Worsening > Improving, 20 s: p < 0.001; 28 s: p < 0.001). Delay differently affected males and females (Fig. 6A, sex × schedule × delay interaction, F_(4, 5603.65)_ = 3.85, p = 0.00395) where females specifically slowed down on the Improving schedule when there was no delay (female Improving > Worsening, 0 s: p < 0.001), but sped up at larger delays (female Worsening > Improving, 20 s: p < 0.001; 28 s: p < 0.001). Females were faster than males on the Improving schedule at the 28 s delay (male Improving > female Improving, p = 0.0067). Males only sped up on the Improving schedule at the 20 s delay (male Worsening > male Improving, p = 0.0091).

The second round of delay discounting was still subject to delay effects on both the small option (Fig. 6B, main effect of delay, F_(4, 50.39)_ = 9.14, p < 0.001) and the large option (Fig. 6B, main effect of delay, F_(4, 53.01)_ = 12.51, p < 0.001). Further experience with delay orientations showed mice mitigated choice response time differences between schedules as they learned to distinguish between the two schedules. Small choice response times had some suggestion of anchoring (Fig. 6B, main effect of schedule, F_(1, 18.60)_ = 10.29, p = 0.0047), but at no specific delays. Large choice response times suggested no anchoring, but delay specific effects (Fig. 6B, schedule × delay interaction, F_(4, 7552.42)_ = 5.25, p < 0.001). Mice slowed down on the Improving schedule during the 20 s delay (Worsening > Improving, p = 0.0189). Females were slower than males when collapsed across schedules (Fig. 6B, sex × delay interaction, F_(4, 53.01)_ = 4.63, p = 0.0027) at the 20 s delay (p = 0.0347) and 28 s delay (p = 0.0094). Mice need to slow down choice time on the Improving schedule to make a large choice when the delay was initially high. Choice time was adjusted to delays in general, but also to the presentation of delays. This indicated increased choice response times are deliberate and are adjusted to not only the cost of the task, but also to the order of the cost.

Schedule effects became more pronounced with extended training. Compared to previous rounds, increased schedule effects were feasible because choice response time tracks cost deliberation according to the order of delays. Mice adjusted response times according to delay on both the small option (Fig. 6D, main effect of delay, F_(4, 51.79)_ = 7.36, p < 0.001) and the large option (Fig. 6D, main effect of delay, F_(4, 45.03)_ = 12.51, p < 0.001). Small choice response times were sensitive to delay according to the schedule (Fig. 6D, schedule × delay interaction, F_(4, 11368.97)_ = 3.65, p = 0.0057) and sex (Fig. 6D, sex × schedule × delay interaction, F_(4, 11368.97)_ = 7.18, p < 0.001). Mice were slower on the Worsening schedule at the 0 s delay (Worsening > Improving, p = 0.0034), female mice specifically were slower on the Worsening schedule at the 12 s delay (female Worsening > female Improving, p = 0.0187). Large choice response times also had specific delay effects (Fig. 6D, schedule × delay interaction, F_(4, 13465.12)_ = 20.00, p < 0.001) with slower response times on the Improving schedule at the 4 s (Improving > Worsening, p = 0.004) and the 12 s (Improving > Worsening, p < 0.001) delays, but also slower response times on the Worsening schedule at the 28 s (Worsening > Improving, p < 0.001) delay. These effects were however sex driven (Fig. 6D, sex × schedule × delay interaction, F_(4, 13465.12)_ = 6.62, p < 0.001) with females being slower on the Improving schedule at the 4 s (female Improving > female Worsening, p < 0.001), 12 s (female Improving > female Worsening, p < 0.001) and 20 s (female Improving > female Worsening, p = 0.0383) delays, and males being slower on the Worsening schedule at the 28 s (male Worsening > male Improving, p < 0.001) delay. Males were significantly faster than females when comparing Improving schedule large choice response times at the 4 s delay (female Improving > male Improving, p < 0.001). Extended experience on delay discounting increased schedule differences in large choice response times. Females in particular slowed down on a schedule that is immediately costly for large choices, which is in line with previous rounds of choice behavior demonstrating anchoring effects are especially prominent in females.

#### 3.5.2. Choice response times reflect increasing reward delivery uncertainty throughout probability discounting

Reward uncertainty can modulate the speed in which choices are made. Assessment of uncertainty can lead to differences in response speed, leading to generally slower choices in situations of greater conflict. A previous report of Worsening probability discounting has demonstrated Long-Evans female rats slowed down choices in general compared to male rats [7,28]. We have previously observed that differences in choice response time can be attributed to individual differences in explorative/exploitative strategy engagement [8,9]. Decision noise contributes to the type of strategy used, something we have demonstrated to change throughout experience with probability discounting. If differences in exploration over training on the probability discounting task reflect increasing conflict as animals better understand the risky cost of a choice, then we should see the emergence of probability-dependent increases in choice response times over our training schedules. Indeed, we found that choice response times tracked the degree of reward uncertainty of each probability block in the most highly trained sections of the task (Probability Discounting III) compared to naive performance of the task (Probability Discounting I).

**Fig. 7.**
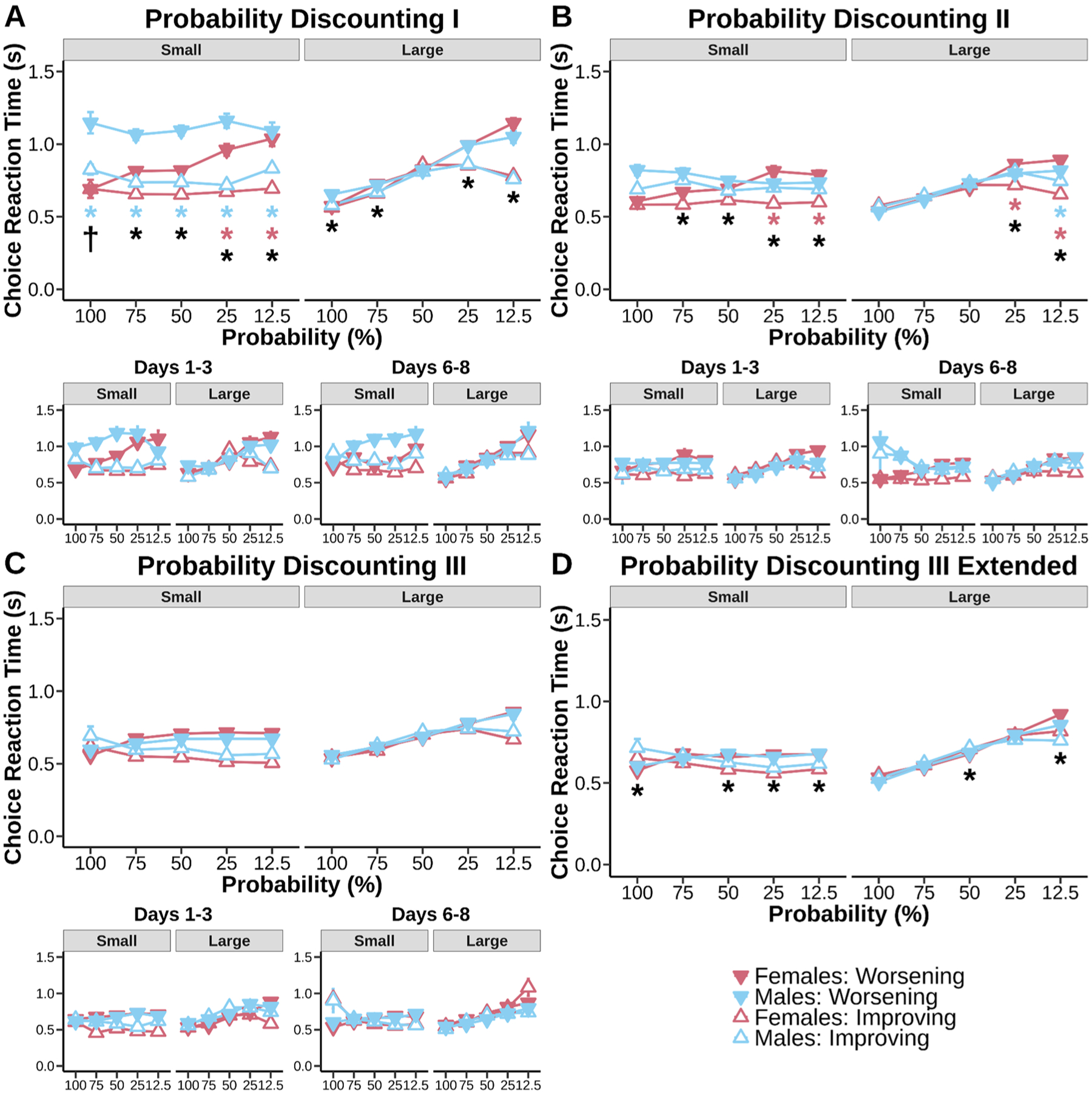
Response times somewhat stabilize but remain sensitive to high uncertainty over probability discounting. Main plots represent a summary of all days of training. Subplots represent day matched periods of training. **A:** As mice first experienced probability discounting, females were slower to respond for small rewards on the Worsening schedule at small probabilities and males were also slower but at all probabilities. Schedule effects appeared for large choice response times across all probabilities except for 50% probability chance of large reward. **B:** Small choice response time schedule effects carried into Probability Discounting II. Mice were consistently slower for Worsening small choices, but females specifically were slower when the probability for the trial blocks were 12.5% and 25% chance for large reward. Both males and females were slower for Worsening large choices at 12.5% probability, but only females were also sensitive at 25% probability. **C&D:** Data in Fig. C represent the first 8 days, data in Fig. D represent all days of training. Sex specific effects at delays diminished with the last round of discounting. General schedule effects at delays were still present. Small choice schedule effects were pervasive for all probabilities of large reward except 75%, large choice differences arose only at 12.5% and 50% probabilities. Figures depict mean ± SEM. Black asterisks indicate significant schedule effects (schedule × delay interactions), black daggers pointing down indicate between sex effects on the Worsening schedule (sex × schedule × delay interactions) of p < 0.05. Colored asterisks indicate significant schedule effects within a sex (sex × schedule × delay interactions) of p < 0.05.

The introduction of a risky uncertain large reward option caused mice to reassess schedule differences. Mice sped up choice response times as the large reward became more probable regardless of whether they were selecting the small reward side (Fig. 7A, main effect of probability, F_(4, 61.21)_ = 4.23, p = 0.0044) or large reward side (Fig. 7A, main effect of probability, F_(4, 61.36)_ = 72.78, p < 0.001). Anchoring was apparent both in small choice (Fig. 7A, main effect of schedule, F_(1, 18.69)_ = 31.53, p < 0.001) and large choice (Fig. 7A, main effect of schedule, F_(1. 13.97)_ = 57.35, p < 0.001). Schedule effects in small choice response times were apparent at different probabilities (Fig. 7A, schedule × probability interaction, F_(4, 7631.93)_ = 3.42, p = 0.0084) for both males and females (Fig. 7A, sex × schedule × probability interaction, F_(4, 7631.93)_ = 3.57, p = 0.0065). General schedule differences for small choice were found at all probabilities except for when the trial block had 100% delivery rate on the large risky side (Worsening > Improving, 12.5%: p < 0.001; 25%: p < 0.001; 50%: p < 0.001; 75%: p < 0.001). Females were slower to make a small choice on the Worsening schedule when the probability of large rewards was at 12.5% (female Worsening > female Improving, p < 0.001) and 25% (female Worsening > female Improving, p = 0.0022). Males were slower to make a small choice on the Worsening schedule at all probabilities (male Worsening > male Improving, 12.5%: p = 0.0031; 25%: p < 0.001; 50%: p < 0.001; 75%: p = 0.0022; 100%: p = 0.0093). Males were slower to make a small choice on the Worsening schedule than females when large rewards were always delivered (male Worsening > female Worsening, 100%: p = 0.0055). General schedule effects for large risky response times were found (Fig. 7A, schedule × probability interaction, F_(4, 61.38)_ = 72.78, p < 0.001). Mice had slower large choice response times on the Worsening schedule at most probabilities except when chance of delivery was 50% (Worsening > Improving, 12.5%: p < 0.001; 25%: p < 0.001; 75%: p = 0.0068; 100%: p = 0.0062). Mice sped up when safety increased within a schedule (i.e. Improving) compared to when risk increased within a schedule (i.e. Worsening) for both small and large options. Males seemed to be most affected by risk orientation while females were sensitive to only the riskiest trial blocks (i.e. 12.5% and 25% probabilities).

Mice were quick to adjust to risky large rewards after Probability Discounting II. As noted by the win-stay/lose-shift data, male mice adapted primarily by shifting to the small choice in a schedule that promotes large initial uncertainty. This is how male mice combat uncertainty and how they made that choice easier (i.e. reduced response time difference). Females dealt with uncertainty by adjusting both win-stay and lose-shift behavior, which created increased difficulty of choice especially when uncertainty was large. Mice responding still slowed down the more uncertain a reward became for large choice (Fig. 7B, main effect of probability, F_(4, 58.53)_ = 62.70, p < 0.001) but not for small choice. Small choice response times were still adjusted according to schedule (Fig. 7B, main effect of schedule, F_(1, 15.46)_ = 13.69, p = 0.0020) but not large choice response times. Mice showed schedule effects for small choice (Fig. 7B, schedule × probability interaction, F_(4, 9006.65)_ = 2.48, p = 0.0422) as soon as the large option became risky (Worsening > Improving, 12.5%: p < 0.001; 25%: p < 0.001; 50%: p = 0.0071; 75%: p = 0.0172). Female small choice response times were especially sensitive to schedule effects (Fig. 7B, sex × schedule × probability interaction, F_(4, 9006.65)_ = 10.14, p < 0.001) at 12.5% (female Worsening > female Improving, p < 0.001) and 25% (female Worsening > female Improving, p < 0.001) probabilities. Mice large choice response times were sensitive at 12.5% (Worsening > Improving, p < 0.001) and 25% (Worsening > Improving, p = 0.0408). Females slowed down on the Worsening schedule when large reward receipt was unlikely (female Worsening > female Improving, 12.5%: p < 0.001; 25%: p = 0.0151), as well as males (male Worsening > male Improving, 12.5%: p = 0.0161). Mice, especially females, showed difficulty in choice when the objective reward value of small choice equaled or was larger than large choice (i.e. 1:1 ratio of reward volume for small choice or large choice only; 25% reward probability).

After extended training on probability discounting, choice response times were no longer sensitive to anchoring. Anchoring in choice was still prominent because probability discounting is a noisy task that mice constantly adjust to, but that aspect becomes less apparent in choice response time. Small choice response times became sensitive to probability again (Fig. 7D, main effect of probability, F_(4, 59.40)_ = 3.22, p = 0.0186) and large choice response times remained sensitive (Fig. 7D, main effect of probability, F_(4, 65.57)_ = 96.56, p < 0.001). Mice were still generally affected by uncertainty for small choice (Fig. 7D, schedule × probability interaction, F_(4, 17455.37)_ = 15.83, p < 0.001) and large choice (Fig. 7D, schedule × probability interaction, F_(4, 33459.35)_ = 41.12, p < 0.001). Mice sped up small rewards on the Worsening schedule when large rewards were guaranteed (Improving > Worsening, p = 0.0038), but slowed down as risk increased (Worsening > Improving, 12.5%: p = 0.0015; 25%: p < 0.001; 50%: p = 0.0062). Mice sped up for large rewards on the Worsening schedule when chance of reward delivery was 50% (Improving > Worsening, p = 0.0101), but slowed down when probability of reward was at its riskiest (Worsening > Improving, 12.5%: p < 0.001). Our results suggest extended experience with probability discounting helped mitigate sex-specific differences in choice response time speed.

### 3.6. Locomotor activity in delay and probability discounting

#### Patterns of locomotor activity are task dependent, but unrelated to changes in choice over experience with the tasks.

It is possible that the shifts in preference for the large reward seen over multiple sessions in these schedules reflect simpler contributions to behavior than changes in decision making processes such as deliberation and cognitive exploration. For example, although animals are familiar with the chamber, perhaps continued locomotor exploration is also changing during this time and influencing the choices animals make. If this were true, then we should see locomotor activity within the chamber changing in synchrony with changes in preference. To test this hypothesis, we measured locomotor activity across all days of testing using infrared beams located at the front (close to the touchscreen) and back (close to the magazine) of the chamber. We did not find support for changes in locomotor behavior that could explain changes in decision exploration over experience.

**Fig. 8.**
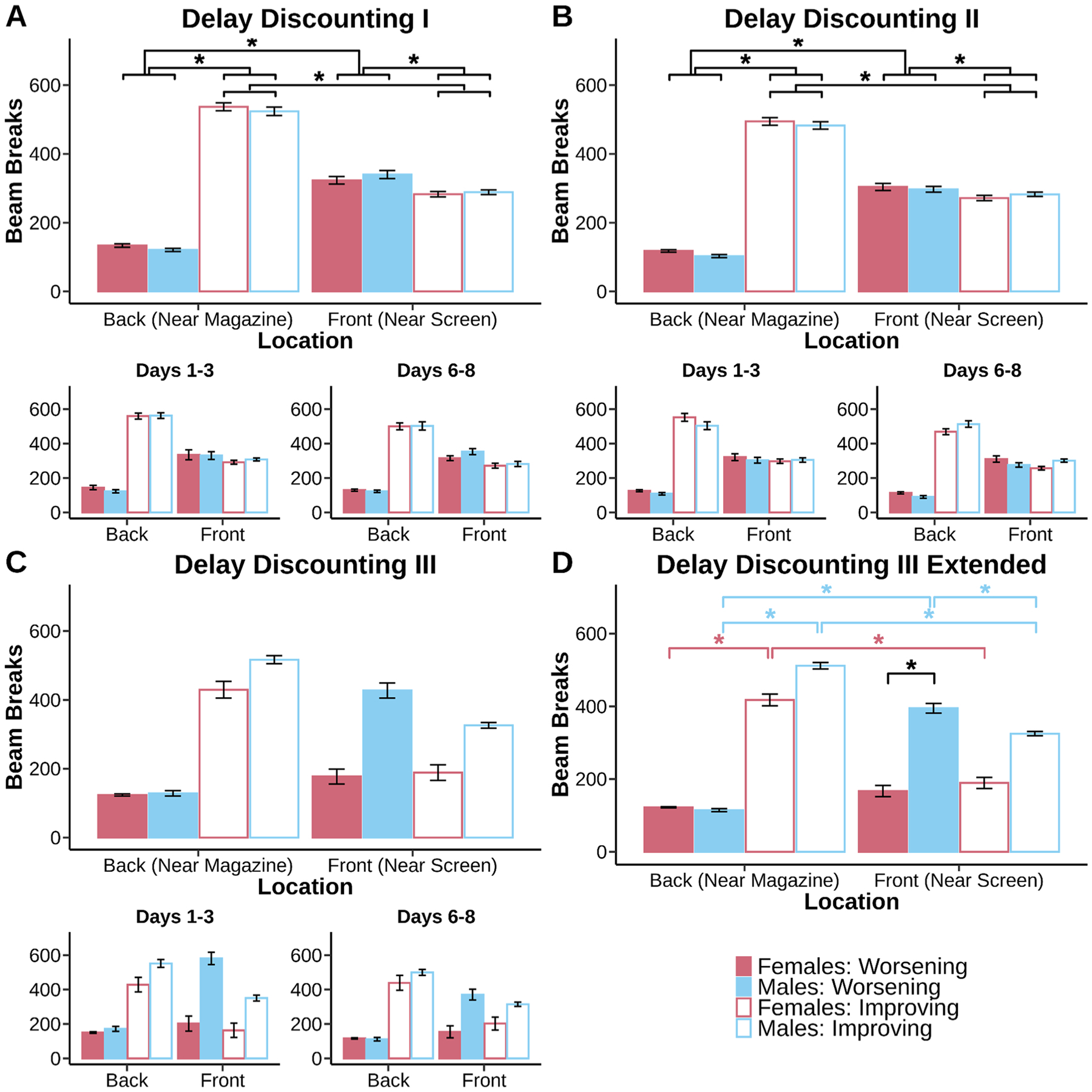
Mice adjust locomotor behavior near the magazine and screen according to the delay discounting schedule. Main plots represent a summary of all days of training. Subplots represent day matched periods of training. Mice broke an infrared beam whenever they crossed to the back of the chamber (near the magazine) or the front of the chamber (near the screen). Shifts in delay discounting schedule orientation changed where mice chose to spend most of their time. **A:** When mice experienced a schedule with increasing delays, exploration of the chamber shifted toward the front of the chamber. However, when the schedule switched orientation to decreasing delays, mice shifted screen preference to magazine preference. **B:** Mice showed a similar pattern of exploration where mice preferred the front of the chamber when delays were Worsening, but shifted that preference to the back of the chamber when delays were Improving. **C&D:** Extended experience with delay discounting caused females to reduce front of chamber exploration regardless of schedule type. Females maintained increased interest in the back of the chamber on the Improving schedule. Males continued to shift their behavior based on schedule type. Figures depict mean ± SEM. Black asterisks indicate significant schedule × break location interactions for Fig. A and Fig. B, but a significant sex effect for Fig. D of p < 0.05. Colored asterisks indicate significant differences within a sex effects (sex × schedule × break location interactions) of p < 0.05.

Over delay discounting, we found evidence of different locomotor strategies between Improving and Worsening versions of the task; however, this did not resolve over time, despite stabilization of choice behavior over that same period. Large temporal gaps between choice and reward delivery at the start of the schedule shifted locomotor behavior from the touchscreen to the magazine. In the first round of delay discounting, mice demonstrated an increase in locomotor activity (Fig. 8A, main effect of break location, F_(1, 430)_ = 3.82, p < 0.001) that was schedule dependent (Fig. 8A, main effect of schedule, F_(1, 430)_ = 21.46, p < 0.001; schedule × break location interaction, F_(1, 430)_ = 34.01, p < 0.001). Mice focused locomotor behavior toward the touchscreen on a Worsening schedule (Worsening front > Worsening back, p < 0.001), but switched focus towards the magazine on an Improving schedule (Improving back > Improving front, p < 0.001). Mice increased activity near the magazine on the Improving schedule compared to the Worsening schedule (Improving back > Worsening back, p < 0.001) and decreased locomotor activity near the touchscreen (Worsening front > Improving front, p < 0.001).

Similar patterns of locomotor behavior persisted for the second and third round of delay discounting. Mice changed their pattern of locomotor activity in a schedule dependent manner (Fig. 8B, main effect of schedule, F_(1, 502)_ = 1119.82, p < 0.001; schedule × break location interaction, F_(1, 503)_ = 1430.63, p < 0.001). Delays again induced large schedule differences which were not reflective of their choice behavior. Locomotor behavior was focused towards the touchscreen on a Worsening schedule (Worsening front > Worsening back, p < 0.001), but shifted towards the magazine on an Improving schedule (Improving back > Improving front, p < 0.001). Mice again checked the back of the chamber more often for the reward on the Improving schedule compared to the Worsening schedule (Improving back > Worsening back, p < 0.001) which came at the cost of decreased activity near the touchscreen (Worsening front > Improving front, p < 0.001). Increased waiting time for the reward caused mice to again narrow locomotor activity near the magazine. Reward delivery delay changes locomotor behavior, but not choice behavior (i.e. anchoring effects are eliminated at this point). Male and female mice started to diverge in locomotor allocation by Delay Discounting III (Fig. 8D, main effect of sex, F_(1, 13)_ = 6.68, p = 0.0226). Mice still altered locomotor activity (Fig. 8D, main effect of break location, F_(1, 916)_ = 13.97, p < 0.001) in a schedule specific way (Fig. 8D, schedule × break location interaction, F_(1, 916)_ = 946.03, p < 0.001). Male and female mice by this point settled on different locomotor patterns (Fig. 8D, sex × schedule × break location interaction, F_(1, 916)_ = 65.07, p < 0.001). Males and females still showed increases in locomotion for the back of the chamber on the Improving schedule (male and female Improving back > male and female Worsening back, p < 0.001), but only males increased their locomotor activity to the front of the chamber on the Worsening schedule (male Worsening front > male Improving front, p < 0.001). Males and females continued to suppress locomotor activity to the front of the chamber on the Improving schedule (male and female Improving back > male and male and female Improving front), but only males demonstrated more interest for the front of the chamber on the Worsening schedule compared to the Improving schedule (male Worsening front > male Improving front, p < 0.001). Males made more beam breaks than females on the Worsening schedule in the front of the chamber (male Worsening front > female Worsening front, p = 0.002). Throughout discounting, locomotor activity patterns remained similar despite choice behavior mice constantly adjusting choice behavior throughout renditions of the task. This suggested locomotor activity was not indicative of learning in delay discounting.

**Fig. 9.**
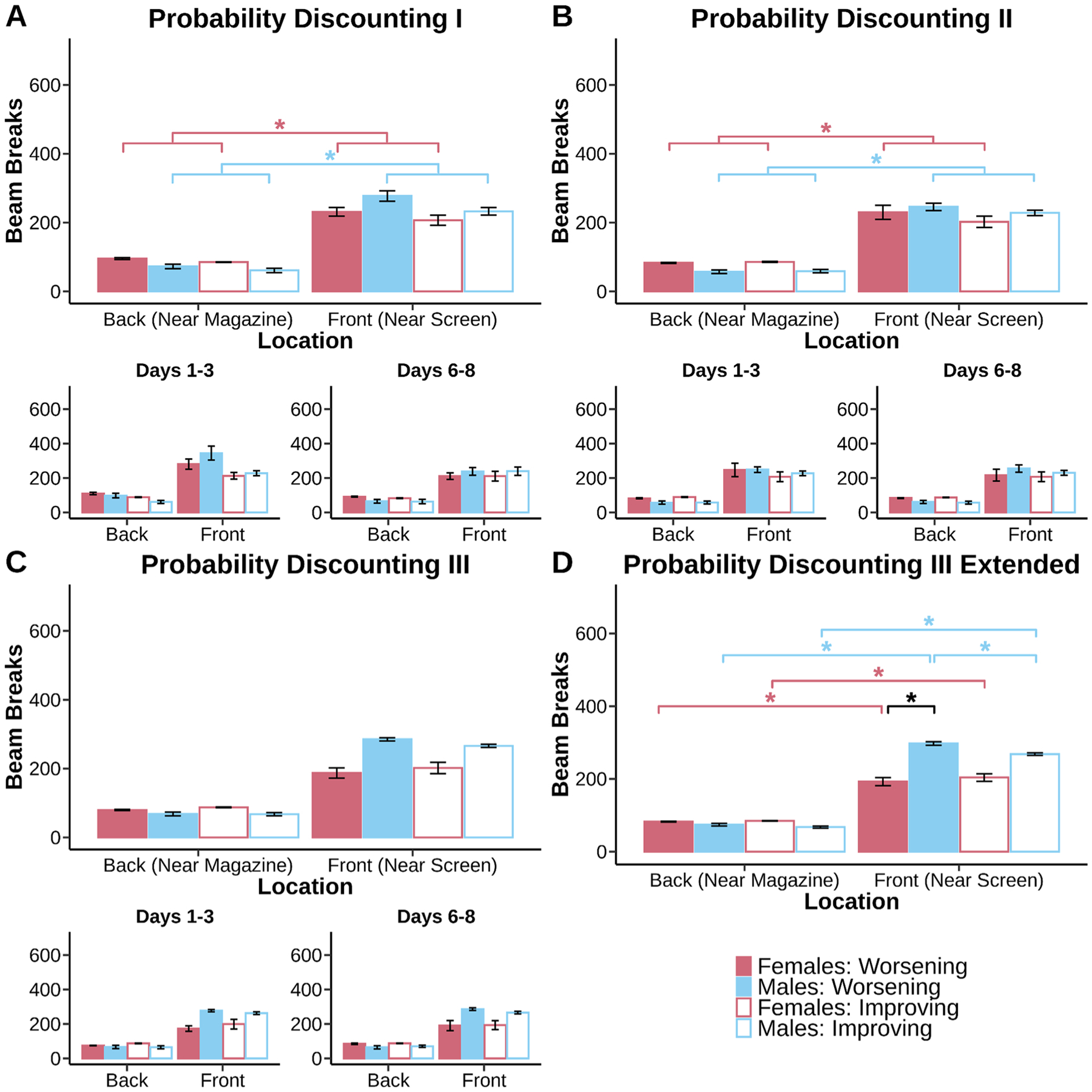
Mice prefer to stay near the screen during probability discounting. Main plots represent a summary of all days of training. Subplots represent day matched periods of training. Movement throughout the chamber is entirely schedule dependent throughout the first round of probability discounting (**A**), the second round of probability discounting (**B**), and the last round of discounting (**C&D**). Male mice however made more beam breaks near the screen on the Worsening schedule compared to females on the Worsening schedule and compared to themselves on the Improving schedule (**C&D**). Figures depict mean ± SEM. Black asterisks indicate significant sex effects of p < 0.05. Colored asterisks indicate significant differences within a sex effects (sex × schedule × break location interactions) of p < 0.05.

In probability discounting, despite constant changes in choice preference there were stable locomotor behaviors across the tasks over the entire testing period, arguing against locomotion as the driver of behavioral change. This was apparent in the first round (Fig. 9A, main effect of break location, F_(1, 446.09)_ = 543.83, p < 0.001). There was some suggestion that mice were sensitive to schedule type again (Fig. 9A, main effect of schedule, F_(1, 446.09)_ = 11.54, p < 0.001), but instead mice experienced general increases in locomotor behavior towards the touchscreen (Fig. 9A, sex × break location interaction, F_(1, 446.09)_ = 11.54, p < 0.001; male and female front > male and female back, p < 0.001). There was no longer a distinction between schedules depending on where the breaks were made.

Mice continued to commit a similar pattern of locomotor changes during Probability Discounting II (Fig. 9B, main effect of break location, F_(1, 450.02)_ = 714.08, p < 0.001). This general increase in locomotor activity again does not give insight into choice, which was influenced by schedule effects (i.e. anchoring). Mice increased locomotor behavior towards the touchscreen (Fig. 9B, sex × break location interaction, F_(1, 450.02)_ = 16.58, p < 0.001; male and female front > male and female back, p < 0.001). Mice generally chose to explore more of the front of the chamber compared to the back of the chamber. Probability Discounting III was characterized by a similar increase in locomotion toward the touchscreen (Fig. 9D, main effect of break location, F_(1, 917.99)_ = 2718.16, p < 0.001). Anchoring at this point was still present. Schedule specific difficulties in choice are not represented in locomotor data. Mice directed behavior towards the touchscreen (Fig. 9D, sex × break location interaction, F_(1, 917.99)_ = 244.96, p < 0.001; sex × schedule × break location interaction, F_(1, 917.99)_ = 7.00, p = 0.0083; male and female front > male and female back, p < 0.001). Male mice shifted their front of the chamber locomotor preference to the Worsening schedule (male Worsening front > male Improving front, p < 0.001). This increase in locomotion for the males on the Worsening schedule was now greater than locomotion for the females on the Worsening schedule (male Worsening front > female Worsening front, p = 0.0073). The volatility or probability discounting reflected in our choice data and computational modeling are not captured by our locomotor activity. Locomotor activity again suggests a consistent pattern of movement in the chamber that is not reflective of the choice data.

## 4. Discussion

We trained male and female mice in a novel battery of delay and probability discounting schedules, in order to assess (1) if mice exhibit stable choice behavior across these tasks, and (2) if sex differences affected stabilization of choice behavior. These tasks are high-priority goals for cross-species translation, and there is some controversy over whether these two tasks test similar or distinct constructs. Overall, we found mice showed substantial discounting, indicating sensitivity to the structure of both tasks. Mice formed stable choice behavior in delay discounting over time, but continued to show anchoring effects throughout probability discounting. To understand why there might be differences in the persistence of anchoring effects between these tasks, we examined win-stay/lose-shift strategies in probability discounting, and across both tasks, fit discounting models to animal data that included both value parameters (*k*/*h*) and an inverse temperature parameter to capture decision noise (β). Win-stay/lose-shift analysis hinted at the presence of schedule-dependent shifts in choice, similar to the anchoring effects prominent throughout probability discounting. Discounting models revealed that mice learned throughout training on both tasks to reduce decision noise. However, the volatility of probability discounting led mice to never really solidify one strategy for the task. Choice response times tracked decision conflict and learning throughout delay and probability discounting, corroborating our findings that changes in choices reflected noisy or exploratory decision making. Our results indicate exploratory decision noise may be underappreciated contributors to behavior in animal models in reward-guided decision making tasks.

Human discounting analyses use value-based models (i.e. *k* and *h* values) to determine the extent of discounting behavior according to the value of the reward. Discounting research has demonstrated how hyperbolic models of value best explain discounting behavior of both humans and animals [22,60]. Discounting steepness is believed to follow a value rule that is liable to change in response to individual factors such as risk tolerance and choice impulsivity [40,49]. Our results are in line with previous research where modeling around a value parameter describes stable discounting behavior [22,39]. However, recent research into animal discounting shows that animals do not always optimize for the discounted value of the reward and implies discounting can arise from multiple individual sources [6,24]. In our data, adding an inverse temperature (β) parameter substantially improved our model fit. There were distinct differences for the role of value in delay and probability discounting. Adjustments in observed behavior in delay discounting were in part captured by experience-linked changes in the value parameter *k*, suggesting that as animals shifted their choices for the large reward over training, they were in fact shifting in their *preference* for that large reward. The same could not be said in probability discounting. Instead, in probability discounting, the value parameter *h* was relatively stable, suggesting that preferences for the large uncertain reward were stable, and that variation in behavior was instead captured by changes in the inverse temperature parameter, suggesting that animals were changing *how much they adhered to their value estimates* when making choices over experience with the task. Our modeling results are in line with our observations of persistent anchoring effects in probability discounting even after extended training, which we interpret as being due to inconsistent adherence to value estimates. These findings are reflective of the volatility with trial by trial reward uncertainty. Our choice to add an inverse temperature parameter to capture decision noise allowed us to better capture adaptations to schedules across tasks, and provide an explanation for differences in choice preference for both tasks. This suggests that decision noise is an underappreciated contributor to value-based decisions in animal models.

Sex effects have not been consistently observed in reward-guided delay or probability discounting tasks [23,41]. Sex differences appear in discounting tasks depending on the type of uncertainty or consequences (especially aversive outcomes; see [42] and [41]), but generally do not appear when the risk is the loss of a reward [23]. Studies have more recently found sex differences in a Worsening schedule of delay discounting [26] and in a Worsening schedule of probability discounting [28] in rats. Previous studies however have noted the possible nuances when relating discounting results across species [60] and strains of rats [28]. Supporting the idea of the importance of genetic background, Bagley et al., [3] have recently demonstrated sex effects in reward guided behaviors are not consistent across mouse strains. Instead, it is genotype background that dictates the strength and direction of a sex effect (sex by genotype interaction). In line with this thought, strain differences in delay discounting have previously been found [25,29]. In the current set of data, animals were able to learn a consistent pattern of behavior to delay regardless of the order of presentation, but probability discounting did not lead to a consistent pattern of choices across sexes. Despite differences in the degree of anchoring across probabilities seen in males versus females in our choice data (Fig. 3), we were unable to capture those effects in our computational model (Fig. 5). Given these findings, we consider three possibilities for why we do not observe sex differences. First, species and/or strain appear to affect the strength of the sex difference, and may play a role here. Second, it is possible that we were underpowered to detect sex differences in decision making given that our lab has observed it in the past [9]. Third, sex differences might be better captured with an additional latent variable not defined within our model.

While delay and probability discounting are often both thought to measure an aspect of choice impulsivity [12,21,31,56], they are found to be weakly correlated even in humans [22,56]. As such, straightforward value models may miss hidden traits specific to delay or risk. In attempting to model these tasks in rodents, one source of variability in reward preference could arise from differences in choice patterns outside of the optimal choice. We included a decision noise parameter in order to capture decision noise hidden in the value parameters [38]. Our results suggest mice not only make value-based decisions, but they also adapt decision noise around the discounted value of a reward. Differences in choices across schedules are better explained by changes in decision noise as opposed to the value parameter. Decision noise is therefore a significant contributor to discounting behavior in our mice, and may be an underrecognized contributor to rodent choice behavior in other contexts.

While we designed our tasks to capture adaptations in value and decision noise, some caveats come with the sequential design of our tasks. Delay and probability discounting rates are significantly impacted by the order of presentation of delayed or probabilistic uncertainties [11,18,50] and could also be affected by whether animals experience delay or probability discounting first [46,53,57]. Fox et al. [18] exposed rats to delays in a Worsening-Improving order similar to what the current set of experiments and found similar anchoring results between schedules. Rats were prone to prefer the large reward when the delays got longer within-session, producing similar anchoring effects as we describe here. Slezak & Anderson [50] however used a random-chance delay exposure paradigm which constantly exposed rats to either order depending on chance. Under this mixed exposure design, schedule differences were mitigated. It is worth noting that our mice did stabilize their performance in delay discounting, despite experiencing two different orders of delay. This could be due to differences in the length of training compared to previous studies like Fox et al. [18]. The order of exposure to delay and probability discounting could account for some of the persistent anchoring we observe with probability discounting. Our delay and probability discounting tasks are structurally similar and thus delay discounting strategies probably influenced choice behavior during at least early probability discounting [37]. We did, however, use a novel house light cue introduced during probability discounting to help mice distinguish between both tasks. Further, each task promotes different types of uncertainty [19] which provides an opportunity for new learning. Our choice data and computational results support these ideas as mice showed differences in adaptation across both tasks. Still, we cannot fully rule out order effects, but this seems less likely given that our behavior and modeling parameter fits indicate that animals continued to adjust their behavior across the duration of the probability discounting task.

Our results support a role for computational modeling in identifying latent variables that contribute to decisions in rodent tasks. As noted above, while *k* and *h* parameters can be used to reflect value in discounting tasks, they are not able to capture the contributions of other variables that might influence choices. Probabilistic tasks, including discounting, are amenable to analyses of choice patterns following wins and losses (e.g. win-stay and lose-shift) but lack a parallel in delay discounting. A global parameter to compare decision noise across tasks is important when assessing choice behavior and testing for factors contributing toward impulsive behavior [12,31,56]. The inverse temperature parameter we included in our computational model helps bridge the gap between the two tasks by allowing for task comparisons and examining influences of order presentation and group differences in valuation of rewards. Our results demonstrate how computational models that account for decision noise are better at detecting different sources of behavioral variability, such as anchoring effects, demonstrated in sequential versions of decision making tasks.
